# Undertaking rapid evaluations during the COVID-19 pandemic: Lessons from evaluating COVID-19 remote home monitoring services in England

**DOI:** 10.3389/fsoc.2023.982946

**Published:** 2023-02-13

**Authors:** Holly Walton, Nadia E. Crellin, Manbinder S. Sidhu, Chris Sherlaw-Johnson, Lauren Herlitz, Ian Litchfield, Theo Georghiou, Sonila M. Tomini, Efthalia Massou, Jo Ellins, Jon Sussex, Naomi J. Fulop

**Affiliations:** ^1^Department of Applied Health Research, University College London, London, United Kingdom; ^2^Research and Policy, The Nuffield Trust, London, United Kingdom; ^3^Health Services Management Centre, School of Social Policy, University of Birmingham, Birmingham, United Kingdom; ^4^Population, Policy and Practice Research and Teaching Department, UCL Great Ormond Street Institute of Child Health, London, United Kingdom; ^5^Institute of Applied Health Research, College of Medical and Dental Sciences, University of Birmingham, Birmingham, United Kingdom; ^6^Global Business School for Health, University College London, London, United Kingdom; ^7^Department of Public Health and Primary Care, University of Cambridge, Cambridge, United Kingdom; ^8^RAND Europe, Cambridge, United Kingdom

**Keywords:** rapid evaluation, reflections, key lessons, COVID-19, mixed methods

## Abstract

**Introduction:**

Rapid evaluations can offer evidence on innovations in health and social care that can be used to inform fast-moving policy and practise, and support their scale-up according to previous research. However, there are few comprehensive accounts of how to plan and conduct large-scale rapid evaluations, ensure scientific rigour, and achieve stakeholder engagement within compressed timeframes.

**Methods:**

Using a case study of a national mixed-methods rapid evaluation of COVID-19 remote home monitoring services in England, conducted during the COVID-19 pandemic, this manuscript examines the process of conducting a large-scale rapid evaluation from design to dissemination and impact, and reflects on the key lessons for conducting future large-scale rapid evaluations. In this manuscript, we describe each stage of the rapid evaluation: convening the team (study team and external collaborators), design and planning (scoping, designing protocols, study set up), data collection and analysis, and dissemination.

**Results:**

We reflect on why certain decisions were made and highlight facilitators and challenges. The manuscript concludes with 12 key lessons for conducting large-scale mixed-methods rapid evaluations of healthcare services. We propose that rapid study teams need to: (1) find ways of quickly building trust with external stakeholders, including evidence-users; (2) consider the needs of the rapid evaluation and resources needed; (3) use scoping to ensure the study is highly focused; (4) carefully consider what cannot be completed within a designated timeframe; (5) use structured processes to ensure consistency and rigour; (6) be flexible and responsive to changing needs and circumstances; (7) consider the risks associated with new data collection approaches of quantitative data (and their usability); (8) consider whether it is possible to use aggregated quantitative data, and what that would mean when presenting results, (9) consider using structured processes & layered analysis approaches to rapidly synthesise qualitative findings, (10) consider the balance between speed and the size and skills of the team, (11) ensure all team members know roles and responsibilities and can communicate quickly and clearly; and (12) consider how best to share findings, in discussion with evidence-users, for rapid understanding and use.

**Conclusion:**

These 12 lessons can be used to inform the development and conduct of future rapid evaluations in a range of contexts and settings.

## 1. Introduction

### 1.1. Summary

This manuscript aims to explore how large-scale evaluations can be conducted rapidly, in tight timescales and with appropriate stakeholder engagement. We aim to show that rapid evaluations in these circumstances can be carried out to a high quality but that sometimes difficult decisions must be made to balance the needs of rapidity with those of scope, rigour, time, and resources.

We begin with a summary of what this manuscript adds to the evidence. We then outline why rapid methods were needed within an evaluation of COVID-19 remote home monitoring services and reflect on key lessons in conducting rapid evaluations.

### 1.2. Background

#### 1.2.1. Why were rapid methods needed within this evaluation?

The COVID-19 pandemic was an unprecedented global event that impacted on and changed the delivery of healthcare services in England and internationally (Hutchings, 2020; Leite et al., 2020; National Health Service, 2020; Oxtoby, 2021) (e.g., healthcare appointments were cancelled or delivered remotely and parts of the workforce were redeployed).

COVID-19 was responsible for millions of hospitalisations and deaths worldwide (Al-Tawfiq et al., 2020; World Health Organisation, 2021). Individuals with COVID-19 sometimes develop “silent hypoxia,” where they have dangerously low oxygen levels but without breathlessness (Greenhalgh et al., 2021). This resulted in patients being admitted to hospital with advanced COVID-19, thus requiring invasive treatment, potential admission to intensive care, and poorer outcomes than if they had been admitted sooner (Alaa et al., 2020; Mansab et al., 2021).

COVID-19 remote home monitoring services were developed internationally at the start of the pandemic to address this clinical concern (Annis et al., 2020; Ford et al., 2020; Karampela et al., 2020; Kricke et al., 2020; Nunan et al., 2020; O'Keefe et al., 2020; Thornton, 2020; Hutchings et al., 2021; Margolius et al., 2021; Vindrola-Padros et al., 2021c). In England, services were rolled out nationally by NHS England and Improvement (NHSEI). Within these services, patients were given pulse oximeters and asked to regularly record and submit oxygen levels and other symptoms to a team of administrators and clinicians *via* digital technologies or over the telephone. Patients were then escalated for further care if necessary (National Health Service, 2021a,b). For an infographic of the service, please see (Nuffield Trust, 2022a).

There was a need for rapid, real-time evidence and learning to support the scale-up and roll-out of remote home monitoring services, in order to respond to the pandemic. Early evaluations of COVID-19 remote home monitoring services in England had provided some evidence on areas such as safety, effectiveness and implementation (Bell et al., 2021; Clarke et al., 2021; Vindrola-Padros et al., 2021b). But there was a need to understand more fully the impact and cost of services, and staff and patient experiences of services, with a view to inform scaling up service delivery and national roll out.

Three studies (see Beaney et al., 2021, 2022; Lloyd and Parry, 2021; Pariza and Conti, 2021 for details of the other two studies) were commissioned to collaboratively conduct evaluations of COVID-19 remote home monitoring services. Within this manuscript, we focus on one of these evaluations, conducted by two rapid evaluation teams: National Institute for Health and Care Research (NIHR) Rapid Service Evaluation Team (RSET) (Nuffield Trust, 2022b) and NIHR Birmingham, RAND Europe (a not-for-profit policy research organisation) and Cambridge Evaluation (BRACE) centre (University of Birmingham., 2022). These centers were commissioned in 2018 to conduct rapid evaluations of healthcare services. BRACE and RSET aim to evaluate new ways of providing and organising care, including impact, cost, implementation and experiences, and to provide lessons for the NHS and care provision (Nuffield Trust, 2022b; University of Birmingham., 2022). The two centers are organised for rapid working as they have multi-disciplinary core teams with standing advisory and public patient involvement groups, with the ability to draw in wider research support or expertise where needed. Since 2018, RSET and BRACE have conducted numerous rapid evaluations of healthcare and social care services (Nuffield Trust, 2022b; University of Birmingham., 2022).

#### 1.2.2. Summary of the evaluation

The evaluation was comprised of three distinct, but closely linked, studies (Phase 1, Phase 2 and care homes study). The Phase 1 findings were used to inform service improvements and national roll-out of services. Research questions and a summary of methods for each phase of the evaluation are outlined in Table 1. Findings from the Phase 1 study (Fulop et al., 2020; Vindrola-Padros et al., 2021b,c), Phase 2 study (Crellin et al., 2021; Fulop, 2021; Walton et al., 2021; Fulop et al., 2021; Georghiou et al., 2022; Herlitz et al., 2022; Sherlaw-Johnson et al., 2022; Sidhu et al., forthcoming a) and care homes study (Sidhu et al., forthcoming a, forthcoming b) have been published elsewhere.

**Table 1:** Research questions and a summary of methods for each rapid evaluation phase.

**Element**	**Length of evaluation**	**Research questions**	**Summary of methods**
Phase 1	Completed within 2 months	1. How have remote home monitoring services been implemented for COVID-19 and what are their main components, processes of implementation, target patient populations, impact on outcomes, costs and lessons learned? 2. What were the characteristics of remote home monitoring models for COVID-19, experiences of staff implementing these models, data processes, staff and resource allocation and lessons learned during wave 1 of the pandemic?	• A rapid scoping review to explore the use of COVID-19 remote home monitoring services (Vindrola-Padros et al., 2021c) • An empirical implementation study of COVID-19 remote home monitoring services in England (in 8 sites) (Vindrola-Padros et al., 2021b)
Phase 2	Completed within one year—data collection took less than 6 months	1. Are COVID-19 remote home monitoring services associated with changes in mortality and use of hospital services? Does the use of tech-enabled oximetry have a measurable effect on mortality and hospitalisations? 2. What were the costs of setting up and running COVID-19 remote home monitoring services and how do these costs vary between tech-enabled and analogue, and analogue-only data submission modes? 3. What are the factors influencing delivery and implementation of COVID-19 remote home monitoring services? Do these vary by type of model, geography, mode of remote monitoring approach (tech-enabled vs. analogue)? 4. What are the experiences and behaviours (i.e. engagement with services, use of other services) of patients receiving COVID-19 remote home monitoring services? Do these vary by type of model, patient characteristics, mode of remote monitoring (tech-enabled vs. analogue)? 5. Are there potential impacts on inequalities? 6. What are the experiences of staff delivering COVID-19 remote home monitoring services? Do these vary by mode of remote monitoring (tech-enabled vs. analogue)?	• Effectiveness studies of COVID-19 remote home monitoring services—we used routinely available data, hospital administrative data and other information produced by the programme to explore impact and effectiveness of services, relating to hospitalisations and mortality (Georghiou et al., 2022; Sherlaw-Johnson et al., 2022) • Cost analysis—We collected aggregated data on patient numbers, staffing models and allocation of resources from 26 sites to explore costs of setting up and running services (NIHR Rapid Service Evaluation team, 2021; Fulop et al., 2021) • National Study of implementation, patient and staff experience in England (in 28 sites)—we conducted documentary analysis, interviews with 5 national leads, surveys with staff leading and delivering services in 28 sites and surveys with patients receiving COVID-19 remote home monitoring services (Crellin et al., 2021; NIHR Rapid Service Evaluation team, 2021; Walton et al., 2021; Fulop et al., 2021; Herlitz et al., 2022; Sidhu et al., forthcoming a) • Case studies of implementation, patient and staff experience in England (in 17 of the 28 sites)—we conducted interviews with staff leading and delivering services and patients receiving COVID-19 remote home monitoring services (Crellin et al., 2021; NIHR Rapid Service Evaluation team, 2021; Walton et al., 2021; Fulop et al., 2021; Herlitz et al., 2022; Sidhu et al., forthcoming a)
Care homes study	Completed within 10 months—data collection took 3 months	1. When and how is pulse oximetry being employed in care homes for managing the health care of residents with COVID-19 and other health conditions? (Including which care home staff are involved in set up, delivery and monitoring and what support care homes receive, whether it is appropriate and weaknesses in providing support) 2. What are the perceived benefits to residents (e.g., health related outcomes, satisfaction with care received, hospital admission evidence, impact on perceived anxiety) of using pulse oximetry in their care home? 3. What are the experiences of staff using oximetry in care homes (barriers, enablers and lessons learnt)? (Including training received, impact of service on staff wellbeing and confidence, challenges faced by care home staff) 4. What are the views of senior care home staff and managers on guidance and resources necessary to support and sustain use of pulse oximetry in care homes? 5. What are the experiences of primary, community and secondary care healthcare staff involved, or supporting use of pulse oximetry in care homes?	• Scoping interviews with NHS leaders, care association directors and care home managers, engaging with relevant literature, co-designing with a user involvement group (Sidhu et al., forthcoming b) • Online survey of care homes in England (Sidhu et al., forthcoming b) • Interviews with care home managers and staff, and with NHS staff who support care homes in England (in 6 sites) (Sidhu et al., forthcoming b)

### 1.3. Literature review and how this manuscript adds to the evidence base

Previous research has outlined what rapid evaluations are, their features, benefits and some of the factors that may support and challenge them (Smith, forthcoming; Vindrola-Padros et al., 2021a; Norman et al., 2022). Important elements of rapid research include: using large multidisciplinary evaluation teams to enable parallel data collection and analysis; different layers of analysis depending on purpose (high level vs. in-depth); feedback loops to share findings while the study is ongoing; building relationships quickly with stakeholders; and piloting data collection tools (Vindrola-Padros et al., 2021a). However, some of the challenges of rapid research include balancing cost and time with rigour and scope and the quality of data (Vindrola-Padros et al., 2021a; Norman et al., 2022).

Building meaningful relationships and coproducing evaluations with key stakeholders are key elements for the development of service innovations and evaluations (Arnstein, 1969; Chouinard and Milley, 2018; Djellouli et al., 2019), with evaluators providing expertise on the methods and process and stakeholders providing context and service specific knowledge (Chouinard and Milley, 2018). A review of stakeholder engagement identified several reasons why stakeholders should be involved in research, including: empowerment, capacity building, increasing the relevance and use of findings and ensuring sensitivity to the specific context (Chouinard and Milley, 2018). Existing evidence highlights that evaluators should identify who should be involved in evaluations, depending on the purpose of the evaluation, and that a range of different stakeholders should be included throughout the process (Chouinard and Milley, 2018). Studies have also highlighted examples of strategies that can be undertaken to engage stakeholders in evaluations, including: the involvement of patient co-investigators, stakeholder advisory boards, patient and public involvement (Kearney et al., 2021); being inclusive; focusing on governance and process management processes; organising gatherings, large-scale events and using creative methods (Chouinard and Milley, 2018). However, findings indicate that it is important to build mutual respect and trust, ensure capacity building, empowerment and ownership, and consider accountability and sustainability of partnerships (Cargo and Mercer, 2008). Within evaluations, tensions between coproducing evaluations and maintaining critical distance, for example designers and implementers of innovations may understandably desire evaluation findings to be positive (Dixon-Woods, 2019). Therefore, maintaining critical distance within any evaluation requires open and frequent discussions regarding the independence of the research and what that means (e.g., findings being published following peer review).

Whilst previous research has highlighted the importance of coproduction and provided examples on how to achieve coproduction during evaluations, further learning is needed on approaches to stakeholder engagement during rapid evaluations, during which the time to build, maintain and sustain relationships is scarce. Additionally, to the authors' knowledge, little research has focused on practical considerations for conducting rapid evaluations, such as project management and administrative support.

This manuscript extends previous evidence by: (i) providing reflections on the process and experience of undertaking rapid evaluation in political and pressured circumstances, and (ii) contributing learning from a large-scale rapid study on how to mobilise mixed-methods rapid evaluations of health care services. Twelve key lessons are outlined which can be used to inform the development and conduct of future rapid evaluations within a broad range of contexts and settings.

## 2. Reflections on conducting rapid evaluations

**Figure 1:**
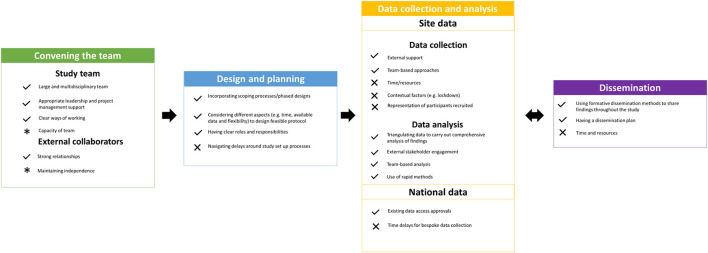
A summary of the things that worked well (denoted by ticks) and challenges (denoted by crosses) when conducting these rapid evaluations. Note: Some aspects were identified as both strengths and challenges [denoted by asterisks (*)]. For example, capacity of the team was both a strength (having a number of researchers providing a percentage of their time to the evaluation meant that we had a larger team) and a challenge (due to having a large number of team members, it was difficult to anticipate how much time each member of the team was needed for the evaluation).

Reflections on conducting rapid evaluations of healthcare services are organised according to five stages of the research process: (a) Convening the team, (b) Design and planning, (c) Data collection and analysis of site data, (d) Collection and analysis of national data, and (e) Dissemination. However, we acknowledge that rapid research often does not follow a linear process, and within this evaluation many of these steps coincided or took place in parallel. Figure 1 shows a summary of what worked well and challenges we experienced within each of these five stages.

### 2.1. Convening the team rapidly

#### 2.1.1. A large and multidisciplinary study team

One of the aspects that worked well within this evaluation was our ability to rapidly mobilise a team which included senior leadership, a project manager and a large number of researchers with capacity to deliver the evaluation. The evaluation was conducted by a large team of researchers from NIHR RSET and NIHR BRACE (Phase 1 included 10 team members, Phase 2 included 15 team members and the care home study included 10 team members), from universities and other research organisations. This pre-existing structure of the two rapid service evaluation teams (NIHR RSET and NIHR BRACE) enabled rapid construction of the project team. The project principal investigator was able to quickly mobilise a multi-disciplinary study team that had expertise in different methods. Team members were selected to ensure that the research team had a broad range of skills and expertise and were from many different disciplines (including data analysis, statistics, sociology, applied health research, health psychology, health economics and project management), and were experienced in conducting politically sensitive, large, mixed-methods evaluations of healthcare services. Team members ranged in seniority from (in academic terms) professors to postdoctoral researchers and research fellows.

The development of the team structure was guided by the rapidity and scope and scale of the evaluation. For example, we began Phase 1 with a smaller team and then expanded the team as necessary once we knew we needed to conduct a larger rapid study. As we needed to rapidly collect large amounts of qualitative data from over 25 sites, the Phase 2 evaluation included multiple qualitative researchers (*n* = 7) who worked as a team to collaborate with external providers, collect and analyze data. The COVID-19 pandemic facilitated the rapid development of our team as some team members had increased capacity to dedicate to this evaluation, due to some other research projects having been paused. Additionally, the research team closely worked with external collaborators (e.g., national stakeholders and local sites) to ensure the success of the evaluations.

#### 2.1.2. Appropriate leadership and project management support

Hands-on management (including principal investigator and project manager leadership and expertise) was needed to support the robust and timely collection and analysis of a large amount of data over a short period of time.

It was important to have support and leadership from an overall principal investigator who had oversight of the whole study and how the different methods fit together, and who kept in active contact with senior members of the evaluation team. The principal investigator needed to skillfully put mechanisms in place to ensure a coordinated and aligned approach. These mechanisms included: attending all project meetings, supporting researchers leading each component, managing each team member, negotiating roles and responsibilities within each sub-group as appropriate, liaising with the wider RSET and BRACE evaluation teams, sharing learning across the three evaluations, developing and managing relationships with external stakeholders, and raising the profile of the study.

Additionally, it was integral to have project management support for many tasks throughout the study. Within the evaluation, project management was provided by a designated project manager instead of researchers. Examples of these tasks included: planning team members' roles, responsibilities, and time commitments on the project, ensuring that the project met internal and external deadlines, planning and arranging a substantial number of meetings for the project each week (including internal team meetings and external stakeholder meetings), constantly reviewing timelines and tasks to ensure that the project was running to time, liaising efficiently with a large number of research project sites and arranging surveys to be printed and distributed.

It is our view that rapid evaluations require more principal investigator and project management time than non-rapid evaluations due to the rapidity of the work, the size of the team, complexities of stakeholder engagement, and the need to balance rapidity and rigour and maintain momentum.

As with any large team, clear but distributed leadership was integral to the success of the evaluation. Within the evaluation, the principal investigator was responsible for leading and managing the overall programme of research, ensuring triangulation of findings and being the point of contact for the funder and national stakeholders. However, day to day leadership was shared amongst the wider team to ensure the success of different aspects of the evaluation. For example, within the Phase 2 study, the quantitative aspects were led by the quantitative researchers, health economic aspects led by the health economist and the qualitative aspects (including ethical approval) were led by one of the qualitative researchers. Within the qualitative workstream, each site had its own research lead and each topic of analysis had a lead researcher. This model of distributed leadership was appropriate in ensuring that each aspect had dedicated commitment to ensuring that it was delivered rapidly and efficiently, ensured that the evaluation succeeded and helped to ensure clear responsibilities and accountabilities.

#### 2.1.3. Clear ways of working together

Whilst this specific team had not worked together before, team members were able to quickly familiarise with each other and mobilise to deliver on this evaluation; supported by the regular weekly online team meetings, clear communication channels (e.g., email, online weekly meetings) and shared values (helped by some team members having worked together previously). Individual researchers were assigned to lead on specific work elements through discussion and agreement in team meetings, ensuring that each component of the evaluation received the time and attention that it required to succeed. There were clear processes outlined for all researchers to follow (e.g., regarding communications to sites, and data collection processes), in order to ensure consistency. Weekly, online team meetings also helped to provide team members with mutual moral and practical support and ensure that the experience was a positive one (particularly as rapid evaluations can be demanding, especially for key individuals involved).

#### 2.1.4. Capacity of team members

Unlike longer-term research studies, rapid studies often end up with researchers providing a percentage of their time to the study rather than one or two dedicated research fellows. This often meant that researchers were juggling several other rapid evaluations at the same time.

There were some challenges relating to difficulties anticipating how much time would be required for each member of the team to conduct the evaluation, continuity of team members and changes in capacity and circumstances. For example, some new team members joined the study for Phase 2, and some additional team members were involved with the care home study. This was challenging as it meant that everyone had slightly different awareness and knowledge about the study initially and needed to be rapidly inducted in the ways of working and project progress so far. However, the overlap of other team members, and the involvement of some team members in all three evaluations, and clear oversight from the project manager, ensured continuity and meant that everyone was able to get up to speed quickly. There were also times when researchers' capacity changed i.e., during times of parental leave, and so the team had to adjust roles and responsibilities to ensure that all aspects of the evaluation were covered, and momentum was maintained. Some of the characteristics of our team that facilitated this rapid evaluation included our rapid evaluation teams having access to a wider pool of researchers that could be drawn on and brought in as necessary, team members being flexible, able to juggle multiple priorities, able to communicate effectively within the team, and willing and able to make rapid decisions; with encouragement and enablement from the principal investigator and project manager.

#### 2.1.5. Establishing a wide network of external collaborators

**Figure 2:**
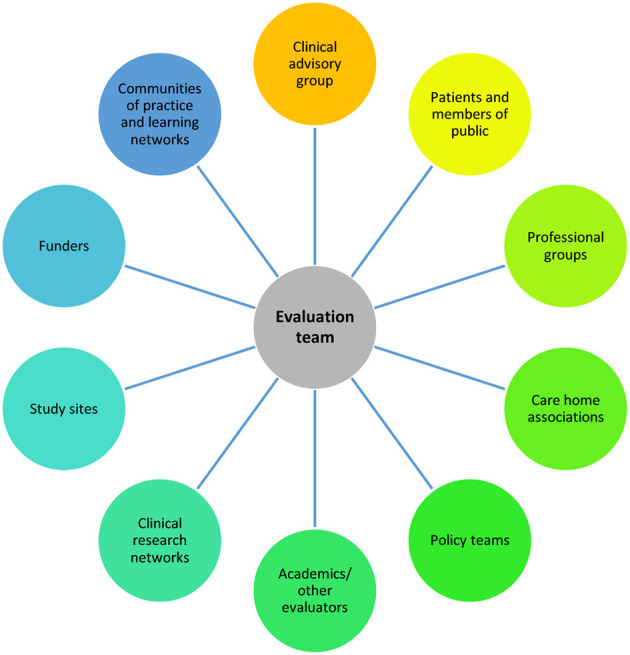
Summary of external stakeholders who engaged with the team.

Within these evaluations, there was a large amount of engagement with external collaborators (see Figure 2 for the groups that we engaged with to design and deliver the study and/or engage as participants).

#### 2.1.6. Strong relationships with stakeholders

The remit of this evaluation was guided (and partly funded) by wider stakeholders, with external clinical collaborators identifying the need for the study early in the pandemic. External collaborators were highly motivated and keen to support the evaluation, and the project was designed with strong collaborations in mind. Stakeholders were motivated to support the evaluation as they were involved in the development and running of the service. Further, stakeholders were keen to build the evidence-base on COVID-19 remote home monitoring services to ensure that they were providing high quality care for COVID-19 patients. Stakeholders also wanted evidence to inform the delivery of future remote home monitoring services within the NHS. In rapid evaluations, there is less time to develop stakeholder relationships, but relationship-building can be facilitated early on by listening to and showing understanding of stakeholders' needs and ensuring these are reflected (as far as possible) in the evaluation. For example, a key focus on exploring inequalities was identified during the evaluation and we adapted our protocol to ensure that this was covered within the evaluation (e.g., within qualitative data collection instruments and amending planned data analysis to include sub-group analysis).

Due to the experience and expertise of the evaluation's principal investigator, some of these collaborations were initiated by the external collaborators (e.g., the Clinical Advisory Group) which comprised individuals with expertise in developing and running COVID-19 remote home monitoring services. The evaluation team also engaged learning networks—networks of local providers and regional and national policy makers who come together to share learning about the development and running of services—which had been set up to support the delivery of services. However, many of the external collaborations were developed during the project, for example, relationships with policy teams, clinical teams, and participating organisations such as associations of care homes.

Given that we planned to conduct primary data collection with staff and patients, we needed to ensure that staff and patients were involved from an early stage to develop an evaluation that would be feasible to implement in practise. However, the rapidity and novelty of the service made it challenging to build a specific public and patient involvement panel that included individuals with experience of COVID-19 remote home monitoring services. Therefore, if studies are to be delivered at speed, there is a need to have pre-existing networks or advisory groups established that can be consulted for rapid advice. We drew on some of our pre-existing structures for this evaluation, developing a Patient and Public Involvement (PPI) panel comprising members from NIHR RSET and NIHR BRACE's PPI panels and these individuals provided advice and feedback at all stages of the project through workshops. Additionally, we sought to obtain additional feedback on study data collection tools from members of the public, with the intention of drawing on experiences of those living with COVID-19. A limitation of drawing on pre-existing networks is that involvement may not include individuals with the exact expertise or experience of the evaluation topic (e.g. those receiving COVID-19 remote home monitoring services).

Relationships with stakeholders were maintained by holding regular meetings, being open and honest about expectations and agreeing what research questions could be answered as part of a rapid evaluation conducted during a period of international crisis, and sharing findings with stakeholders in formats appropriate to them throughout the evaluation.

Within the evaluations, stakeholders were continually engaged and motivated, perhaps due to the urgent nature of COVID-19. Additionally, we sought to avoid stakeholder fatigue by collaborating with national and local stakeholders to find out appropriate and undemanding ways of engaging them within our study, sharing findings and discussing the study with them.

#### 2.1.7. The importance of maintaining independence

As with non-rapid studies, there is a need to balance engaging stakeholders through building trust, whilst maintaining the independence of the research. Maintaining independence when evaluating healthcare services can be challenging due to optimism bias of programme designers/implementers (Dixon-Woods, 2019). As with non-rapid studies, researchers need to navigate sharing potentially “less desirable” findings arising from evaluations and retain their independence throughout the evaluation. This may be particularly important in rapid evaluations in which the topic and findings may be potentially politically sensitive—e.g., because there may be an understandable organisational or political desire for evaluation findings to be positive—and there has been less time to develop relationships. Therefore, these discussions should take place as soon as possible within rapid evaluations.

### 2.2. Design and planning

#### 2.2.1. Building scoping work and phased approaches into design

Our study was intentionally phased in design (beginning with Phase 1 to inform Phase 2 and then being extended to care homes). The Phase 1 study was co-designed with our clinical advisory group and communities of practise set up to support and share learning between those leading and delivering the service. It was also informed by a 4-week scoping exercise which included an initial scoping of the literature, discussions with a small number of sites, documentary analysis, understanding what data were being collected and how they were being used, and discussions with external stakeholders.

The focus on scoping early on, and the phased evaluation approach, helped with the design and development of later stages of the study (including shaping goals, aims and methods of later stages). For example, Phase 1 in and of itself could be seen as an extension of the scoping work. Additionally, conducting a scoping process revealed that relevant literature was scarce on the use of pulse oximeters in care homes, especially when this sector was adversely affected by COVID-19, and identified evidence gaps. This motivated the care home evaluation team to plan expert interviews to find out more about pulse oximetry in care homes, and work with locally set up remote home monitoring models.

#### 2.2.2. Designing feasible protocols for rapid evaluation

We developed the protocols for each of the phases within the evaluation, building on the scoping process and learning from previous phases. The protocol for our Phase 2 study built on our learning from Phase 1, specifically the need to focus on outcomes and patient experience, and informing the sampling approach, and was developed with input from our Clinical Advisory Group and other research teams working in the area. The protocol for the care home extension to the evaluation drew on the Phase 1 and Phase 2 protocols. The protocols were developed by the whole team involved in the evaluation but with individuals taking the lead on different workstreams depending on their skills and expertise.

When planning each stage of the evaluation, we carefully decided on our methods and the scope and scale of each study depending on the timescales of each stage. For example, in Phase 1 we did not include patients due to the timescales needed to obtain the necessary approvals and plan and collect data. Additionally, in the care home study we did not include residents, for various reasons including: logistical challenges collecting data, rapidly ensuring residents' capacity to share views and experience, difficulties collecting data remotely due to sensory (visual/hearing) or speech impairments, lack of feasibility of methods such as in person interviews or non-participant observations (given the pandemic restrictions), and the need to carefully pilot data collection tools. This demonstrates the trade-offs between rapidity and the scope of evaluations.

We designed a methodology for the effectiveness evaluation that would use data we anticipated would be possible to obtain rapidly or where existing arrangements were already in place: existing national datasets, aggregated public health and service data, and patient-level hospital data (which we held and had existing permissions to use through an existing contract with the NHS). We steered away from planning to use new patient-level data on the use of COVID-19 oximetry services, as these data would take longer to become available. Our intention was to provide emerging findings that would add value to the service before the more robust analyses using patient-level data were available. Our analysis approach was to use aggregate level data at an area level: relating mortality and use of hospital resources to the level of enrolment to the programme within the area. Similar methodological approaches were used to evaluate the effectiveness of COVID-19 virtual ward services (for those discharged early from hospital).

When developing the protocols and designing the evaluations, there were many uncertainties (e.g., the service and accompanying documentation were rapidly evolving, lockdown restrictions were changing rapidly and the quality of service data was uncertain), therefore the team needed to build flexibility into the research proposal and ethics application. Examples from our study included offering sites flexibility in the method that they used to recruit participants, and offering both online and paper surveys (the latter using freepost envelopes). The team also had to be flexible in iteratively developing the protocol and data collection approaches to take changes to the COVID-19 remote home monitoring national programme (National Health Service, 2021a) into account (e.g., changes in eligibility criteria and terminology used). We were also unsure about exactly what the data being collected by the new services was going to look like (e.g., what level of detail would be recorded), so we had to be flexible regarding the type of economic analyses that we would be using. Additionally, it was difficult to anticipate the exact focus of all of our analyses, as some became necessary/feasible only part way through the analysis (e.g., findings relating to inequalities and implementation in comparison to national standard operating procedures).

#### 2.2.3. Navigating study set up processes

The evaluation was identified as a priority by NIHR during COVID-19, which facilitated the speed of ethical approvals, set up, data collection and subsequent amendments (needed due to ever changing COVID-19 restrictions and evolving nature of the service). However, even with fast-track approval processes, we still encountered delays in local governance approvals (e.g., getting study sign-off at each of the 28 Phase 2 sites). Additionally, it took time to gain access to sites and communicate with gatekeepers who were understandably prioritising clinical issues.

What worked well when setting up the study was: distributing responsibilities for following-up different sites among the research team, asking for support from university departments, engaging local research and development offices at participating sites, and requesting support from Clinical Research Networks, which can provide practical data collection support for researchers in England.

#### 2.2.4. Clear roles and responsibilities facilitating set-up

Within the team we set clear roles and responsibilities. Different team members took the lead on different topics. For example, for Phase 2, we had two team members working on the effectiveness aspect, three members working on the cost analyses and a larger team of researchers working on the qualitative workstreams. For the qualitative workstreams, having lead researchers for different study sites ensured that researchers had time and capacity to follow up local approvals with their sites. However, this may also add a risk if researchers are unexpectedly unavailable. It was important to ensure good communication between leads and to have back-up plans in case of issues. Within rapid studies, flexible team working and strong communication between team members are vital in case people's work needs to be covered at short notice (and where pausing an element of a study is not feasible due to time constraints).

### 2.3. Data collection and analysis of site data (interviews, surveys, cost)

Across the three phases in the evaluation, we rapidly collected a large amount of data directly from sites (see Table 2).

**Table 2:** Summary of primary data collected across the three evaluations.

**Evaluation phase**	**Number of sites**	**Length of data collection period**	**Number of survey responses**	**Number of interviews**	**Number of sites responding to cost survey**
1	8 sites	2 months	N/A	– 22 staff	7 sites
2	28 sites	< 6 months	– 1,069 patients/carers – 292 staff	– 62 patients/carers – 58 staff – 5 national leads	26 sites
Care homes	6 care homes (interviews) and a national survey	3 months	– 232 care home managers	– 31 staff – 3 national level staff	N/A

**Figure 3:**
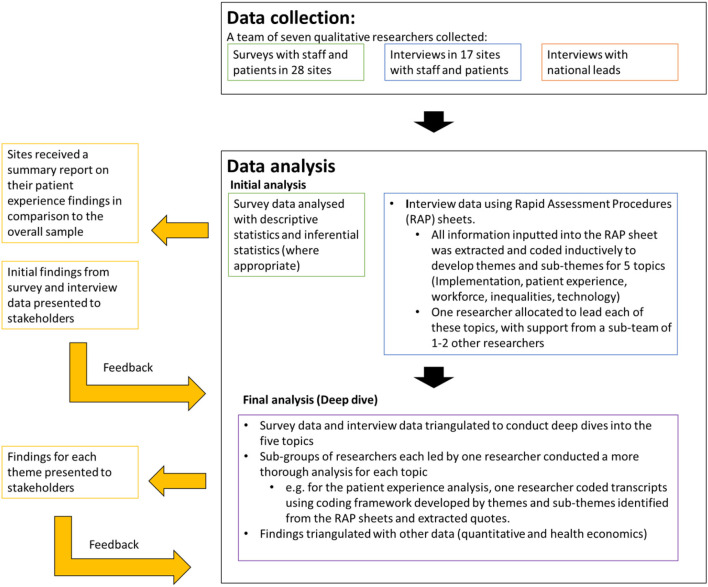
Data collection and analysis processes, together with feedback loops for the Phase 2 evaluation.

To illustrate how data were collected and analysed, and to give an example of how feedback informed the findings within our study, Figure 3 demonstrates the data collection and analysis process for the Phase 2 COVID-19 remote home monitoring evaluation.

#### 2.3.1. External support

Support from our wider networks and external stakeholders facilitated data collection. For example, we presented at national and local meetings, and this enabled us to recruit a sufficient number of sites for the Phase 2 project. Support from the Care Quality Commission and from associations of care homes enabled national distribution of the care home survey. In Phase 2 of the main study, each of the sites had members of staff who took a coordinating role and were crucial in supporting with the recruitment of patients, carers and staff for interviews and sent out surveys. The evaluation was mutually beneficial as we provided sites with summaries of feedback from the patient survey. Similarly, for the care homes study, many social care organisations facilitated survey recruitment by sending out surveys and encouraging responses from care homes and the Care Quality Commission provided a link to the survey in their fortnightly newsletter to all registered care homes, which meant that we could achieve 100% coverage rapidly and at low cost. Without motivated and driven stakeholders, who were passionate about finding out whether services were working and benefitting patients, the evaluation would not have been successful.

#### 2.3.2. Team-based approach

Our team-based approach for data collection meant that we were able to rapidly collect interview data across multiple sites. All lead researchers were responsible for conducting an initial scoping meeting with service leads at their sites, liaising with study coordinators regularly regarding recruitment, data collection and response rates. This approach helped us to understand the processes of each site in a thorough way and build relationships.

#### 2.3.3. Time and resources

Time and resources were a challenge for our rapid evaluations. Managing recruitment and data collection across a large range of sites was time consuming and required a large team and access to resources, for example, the ability to print and deliver large numbers of paper surveys and return envelopes. One challenge we accounted was that we did not know how many paper survey responses to expect, and consequently what level of resource would be required for physically collecting surveys and entering the data from them into the system. This uncertainty also placed additional demand on the resources and time that NHS staff needed to mail out surveys.

#### 2.3.4. Contextual factors—The role of technology in enabling rapidity

We had to overcome challenges resulting from government restrictions in response to the pandemic, for example, during lockdown researchers were unable to travel into the office to access postal survey responses. We used technology to collect data wherever possible, including using Microsoft Teams, Zoom and telephone for interviews, and conducting electronic surveys with staff, patients, and care homes, and providing electronic information sheets and consent forms wherever possible. We were mindful though that not everyone can access electronic materials, and so we also allowed for paper-based patient surveys (with freepost envelopes) and provided the option for information sheets and consent forms *via* post where needed. Having access to REDcap (an online survey tool), which was linked into the university's secure survey platform, supported rapid data collection. The online survey took time to set up initially but then sped up data collection and analysis. Additionally, conducting interviews remotely enabled more rapid data collection of interview data, as we were able to conduct multiple interviews in a short space of time, without the need for travel for researchers, or unnecessary disruption to participants' clinical or operational work.

#### 2.3.5. Representation

Although we developed our study to ensure wide representation, as with many other studies, we had challenges recruiting a wide range of participants, we experienced low response rates on surveys, and we found it difficult to recruit patients and carers to interview who did not receive the service or had disengaged from the service. Our participants were under-representative of some groups, e.g., some ethnic minority groups, despite using strategies to increase representation (e.g., paper surveys and translated surveys). Whilst surveys were available in six languages other than English, there was no uptake of these translated surveys. Further strategies could have been taken to ensure representation, such as including summaries of the study in different languages, to allow participants to request the survey or interviews in another language, or working with specialists to ensure representation of groups that were not represented within our sample (Farooql et al., 2018). However, due to the rapid timeframe of our study (< 6 months for data collection within Phase 2), we were unable to achieve this. Challenges associated with achieving representation were considered during analysis and dissemination.

#### 2.3.6. Triangulation

We were able to triangulate data across different workstreams and different evaluations to provide a comprehensive as well as rapid picture of the development, coverage, implementation, effectiveness, and cost of remote home monitoring services for COVID-19. For example, we were able to use qualitative findings to help interpret our findings relating to cost and effectiveness (e.g., reasons for low enrolment rates and the large variation in service implementation). Additionally, we were able to compare and contrast findings from across different phases (e.g., the finding that services differed markedly across the country was supported by findings from the scoping review and the implementation study from phase 1).

#### 2.3.7. External stakeholder engagement

Throughout the analysis phase, we held workshops with external stakeholders to discuss and shape analysis and to provide formative feedback. This helped us to share findings rapidly throughout the analysis process, refine and ensure validity of our analysis, and discuss any potentially challenging or ambiguous findings early on in the process.

#### 2.3.8. Team-based analysis

Team based analysis was crucial for rapidly analysing the large amount of site data produced within this study. We held regular meetings and workshops with the whole team to discuss and shape interpretations of findings. Having a large team of 7 qualitative researchers within the Phase 2 remote home monitoring study enriched data analysis, as different researchers (together with a sub-team of 2–3 researchers) were able to take the lead on “deep dives” of different analysis topics, including patient experience, inequalities, workforce, technology and implementation. Different members of the team took responsibility for addressing different research questions, and each lead researcher then worked with a smaller team of researchers to conduct the analysis and write up emerging findings. Despite sub-teams taking the lead on specific analyses, all researchers had the opportunity to contribute to the analysis and share comments. This meant that other researchers were able to pick up and continue analysis when researchers were unavailable or busy with other work. Using a team-based approach also enabled us to get to the findings more quickly, as our approach involved regular cycles of collective sense-making and interpretation, rather than the traditional linear (transcribe, code, individual analysis, mapping/charting of findings) approach. Therefore, without a team-based approach, it would have been difficult to conduct the analysis within a rapid time frame.

#### 2.3.9. Use of rapid methods

We used rapid assessment procedures (Vindrola-Padros et al., 2020) (tools and forms used to rapidly capture key findings from different data sources) to analyze qualitative interview data. Using these forms, we highlighted summary findings from each data source for each site. This enabled us to draw the findings together from across different types of interviewee (e.g., different types of staff, or patient/carer interviews) much more quickly, thus arriving at our interim findings much more quickly. Some team members had prior experience of working with rapid assessment procedure (RAP) sheets and consequently knew that they would be appropriate within the rapid timescale. Within the evaluation, we used RAP sheets to add notes and summaries of findings from different interviews for each site. We then coded the findings inputted into the RAP sheets and developed themes and sub-themes (Vindrola-Padros et al., 2020). This worked well as it enabled us to make note of key findings throughout the data collection process, share key findings between ourselves, and conduct analysis rapidly. However, we found it challenging at times to get the right balance of detail of information inputted into RAP sheets (with different researchers inputting different levels of detail). This at times made it necessary to go back to the transcripts for clarification or conduct further analysis.

For Phase 2, we were able to use a layered approach to analysis: high level rapid findings then followed by in-depth deep dives. For example, from the high-level analysis using the RAP sheets, the team was able to identify emerging issues that warranted further investigation. Once we had identified the issues warranting further investigation, we went back to the “raw” data (*via* coding transcripts) to explore these issues. Given the large team approach, we were able to do this within the rapid timeframe, strengthening the analysis.

Whilst the qualitative parts of the study drew on theoretical frameworks and previous literature, we took a layered approach to analysis. Therefore, the analysis was not entirely structured around these frameworks. Initial analyses were informed by empirical literature, but then we applied different and appropriate theoretical frameworks in the various in-depth analyses which followed. This was in part due to the rapid timeframe, evolving nature of the focus of the evaluation and because we did not specify how these frameworks would be used when rapidly developing the protocol. There is scope for further research into how theoretical frameworks can efficiently be used in rapid evaluations; as this reflection is consistent with previous research which indicates that the use of theoretical frameworks is often limited in rapid evaluations (Vindrola-Padros et al., 2021a).

### 2.4. Collection and analysis of national data

To assess the effectiveness of the services, national data on what was known about the delivery of COVID-19 oximetry services was combined together with data on COVID-19 incidence and mortality, and routine hospital data (Georghiou et al., 2022; Herlitz et al., 2022). The hospital data came from Hospital Episode Statistics (HES) and was the only source used that was at patient level. We already had access and permissions to use the hospital data for NIHR RSET evaluations, and we also set up data sharing agreements with Public Health England and NHS Digital to allow us to use aggregated data that were not publicly available. Two data collections relating to implementation of the service were new: one reported numbers of people enrolled on the remote monitoring programme in the community and the other reported the numbers of patients discharged to remote monitoring after a hospital stay. Throughout the Phase 2 study, we attended weekly evaluation data meetings with the NHS and all the evaluation partners; these helped us to coordinate plans, understand the new datasets being collected, and to gain rapid access to them.

Because we were using aggregated data and could not follow individual case histories, we had to make a number of assumptions, for example, about the time lags between the initial diagnosis of COVID-19, enrolment to the oximetry programme and outcomes (admission to hospital or death). Any uncertainty that resulted from this was explored with sensitivity analysis whereby we investigated the relative impact of changing these assumptions.

This evaluation indicates that it is possible to use aggregated data rapidly to evaluate services (with caveats) and, while there are risks with relying on new, bespoke data collections for rapid evaluations, simultaneous site-level collections can help to validate new data collections where quality and completeness of data are uncertain.

### 2.5. Dissemination

Throughout the project, we consulted with stakeholders on how best to share findings which would allow them to quickly make sense of them and apply these findings to the development of the remote home monitoring services in the most impactful way. Channels for disseminating research findings were discussed with stakeholders (national and local) throughout the study to ensure that findings were presented in a format that was most useful to relevant stakeholders and target audiences. Agreed dissemination methods included providing formative feedback to stakeholders through meetings and analysis workshops, the use of slide packs to share emerging findings. These methods were complemented by other methods (including formal written reports).

Dissemination channels included:

- Peer reviewed journal articles and preprints (Crellin et al., 2021; Greenhalgh et al., 2021; Vindrola-Padros et al., 2021b,c; Walton et al., 2021; Georghiou et al., 2022; Herlitz et al., 2022; Sherlaw-Johnson et al., 2022; Sidhu et al., forthcoming a).
- Slide sets (Fulop et al., 2020; NIHR Rapid Service Evaluation team, 2021; Imperial College London, 2022).
- Final reports for the funding body (Fulop et al., 2021; Sidhu et al., forthcoming b).
- Blogs/news articles (University College London, 2020; Vindrola et al., 2020a,b; Sidhu, 2022; Walton and Fulop, 2022; Yahoo! Finance, 2022).
- Videos (NIHR BRACE, 2022).
- Infographics (NIHR BRACE and NIHR RSET, 2022; Nuffield Trust, 2022a).
- Presentations of interim and final findings to policy, clinical and academic audiences.

Sharing interim findings throughout the project has been beneficial in ensuring that the findings can be useful to stakeholders and used to inform future service developments. Findings from Phase 1 were used to inform the decision to nationally roll out services. Findings from all three phases were disseminated widely. A lot of our dissemination was enabled by existing relationships with external stakeholders and by the team being visible and involved in national, regional, and local networks or events. Producing a wide range of different dissemination outputs ensured that our findings reached a range of audiences.

One challenge was balancing time and resources with dissemination, as producing interim findings and outputs for a wide range of audiences can take time and can take away from producing outputs such as peer reviewed publications. However, this was balanced by implementing a publication strategy (i.e., scheduling papers and outputs, with lead author teams, in parallel with the final report). This publication strategy enabled us to produce outputs in a timely manner, ensured that the team had clear goals and deadlines in relation to different dissemination activities, and that each dissemination output had someone leading on it. However, gaining feedback on draft outputs from a large range of stakeholders involved in the evaluation does take time and may risk delaying final outputs. Given the time involved in disseminating findings in different ways, we prioritised dissemination to ensure that stakeholders and funders received interim findings prior to more formalised publications.

## 3. Key lessons

Drawing on these reflections, we have developed twelve key lessons for researchers and commissioners to consider when conducting large scale rapid mixed-methods evaluations of healthcare services in future (see Table 3). Lessons are grouped into four themes: (i) rapidly working with stakeholders, (ii) feasibility of rapid evaluations, (iii) rapid methods and (iv) team characteristics and management for rapid evaluations. Below, we discuss potential challenges associated with each recommendation.

**Table 3:** Key lessons for conducting rapid evaluations of healthcare services.

**Theme**	**Lesson for conducting large-scale mixed-methods rapid evaluations of healthcare services**
Rapidly working with stakeholders	1. Building relationships with external stakeholders rapidly is challenging—need to find ways of building rapport and trust quickly (e.g., open conversations)
Feasibility of rapid evaluations	2. Consider the needs of your rapid evaluation and the resources that will be needed to achieve this
	3. Rapid studies need to be highly focused, and scoping work is critical for making decisions about what to include (and what to exclude/omit) and what approaches for quantitative analysis to adopt
	4. Not everything can be done rapidly; teams need to carefully consider and explain what cannot be done when the timescale is short. Evaluations should have focussed and specific research questions which are explicitly relevant to addressing a policy or practise issue
	5. Structured and standardised processes foster a consistent approach and allow work to be quickly picked up by new or other team members if needed
	6. When working rapidly, there is a need to be responsive to changing needs and circumstances, therefore the study needs to be planned to allow flexibility
	7. Consider the risks associated with new data collections of quantitative data and their usability
Rapid methods	8. Consider whether it is possible to use aggregated quantitative data, and what that would mean when presenting results
	9. Consider using structured processes and layered analysis approaches to rapidly synthesise qualitative findings
Team characteristics and management for rapid evaluations	10. The quicker and more multidisciplinary the study, the larger the team that may be needed and the more robust the leadership, oversight and management of the team that will be required
	11. Ensure that all team members know their roles and responsibilities and have ways of clearly communicating (with clear goals in mind when doing so) with other members of the team, to ensure that the project continues to progress rapidly
	12. Don't slow down or wait when it comes to dissemination. Think about how best to present findings as early as possible so that they can be understood and used quickly (e.g., to make decisions)

### 3.1. Rapidly working with stakeholders

#### Lesson 1: Building relationships with external stakeholders rapidly is challenging—Find ways of building rapport and trust quickly

Rapidly building relationships with a range of external stakeholders (including policymakers, those involved in developing and delivering the service nationally and locally, research departments, and patients and/or carers) is crucial to the success of a rapid evaluation. Yet, building relationships with external stakeholders rapidly can be challenging. Researchers working on rapid studies should see relationship building as a key activity and invest time in it throughout the study, even if it may seem to slow down the pace of the study. Some ways of building rapport and trust quickly include: consistently showing up to meetings to demonstrate commitment to show this is our priority as well as theirs; showing that the research team understands the stakeholder's priorities and concerns; listening to their advice; being flexible; delivering outputs on time; sharing early thoughts on the proposed design of the study; and promptly sharing study findings.

Building trust must be balanced with the need to make explicit the objectivity of the research team and a distinction between being answerable to funders but remaining aware of the interests and priorities of policy makers. The need for critical distance and researcher independence should be agreed upfront and maintained throughout the project. For rapid studies, it is particularly important to have open and honest conversations with stakeholders to agree ways of working (e.g., how often will you meet), to discuss and agree on terminology, and about expectations and the independence of the evaluation, is critical to ensure that all parties of the evaluation know what to expect and their role within it. As with all evaluations, it is important to obtain sign up from stakeholders and evidence users regarding the independence of the findings and that findings will be published following peer review, regardless of the direction of findings. However, within rapid evaluations, these relationships need to be built more quickly. Independence and critical distance are facilitated by the receipt of independent research funding.

Within rapid evaluations, it is important to be clear on who liaises with external stakeholders to ensure efficiency and rapidity of collaborations. For example, within the COVID-19 remote home monitoring study, the principal investigator was the main point of contact with national stakeholders (policymakers and funders). Meetings were attended by the principal investigator and lead researchers. All the local sites taking part in the study had a lead researcher who was their primary contact and who met with them to discuss the study. Two researchers were responsible for liaising with the patient and public involvement panel throughout the evaluation.

### 3.2. Feasibility of rapid evaluations

#### Lesson 2: Consider the needs of your rapid evaluation and the resources that will be required

Due to the compressed nature and the need to work to stipulated (often short) timeframes, rapid studies are not necessarily “cheap”! Large-scale rapid evaluations can be resource intensive, requiring more researcher time and hence more funding than initially expected. It can be challenging to fully anticipate upfront exactly how long certain activities will take (e.g., setting up research sites locally), and how many resources will be needed. It is important to allocate sufficient time and resources to ensure that the evaluation is completed in the desired timeframe.

#### Lesson 3: Rapid studies need to be highly focused, and scoping work is critical for making decisions about what to include (and what not to include) and which approaches to adopt for both qualitative and quantitative analyses

This manuscript, together with previous research (Vindrola-Padros et al., 2021a), highlights that scoping work is key to any rapid evaluation. Scoping work and/or phased designs help to identify the context and support the development of a protocol that can be feasibly conducted within rapid timeframes. The scoping work, stakeholder engagement and earlier phases of the research can help you to decide what is appropriate and possible within your evaluation. This is particularly important for quantitative aspects of an evaluation where impacts of a new service may not be seen over the time available or obtaining permissions to access or link specific data sets can be a long process.

#### Lesson 4: Not everything can be done rapidly; teams need to carefully consider and explain what cannot be done when the timescale is short. Evaluations should have focused and specific research questions which are explicitly relevant to addressing a policy or practise issue

Some research questions and designs do not lend themselves to rapid evaluation. In our studies we had to make decisions about whether, for example, to include interviews with residents of care homes within our study; and this was not felt to be feasible within the rapid timeframe we had. When planning a study, it is necessary to consider what approvals are needed and how long approvals may take and make pragmatic decisions. This can inform the design of the study and ensure that the rapid evaluation is not unduly delayed. Evaluations should have focused and specific research questions explicitly related to addressing policy or practise issues within a rapid timeframe.

#### Lesson 5: Structured and standardised processes foster a consistent approach, and allow work to be quickly picked up by new or other team members if needed

For rapid evaluations conducted by a large team, standardised processes are crucial to ensure a consistent approach between team members, for example, templates of site emails, documented procedures for liaising with sites, spreadsheets documenting key contact or decision points with sites. The other benefit of using structured approaches is that they allow work to be quickly picked up by other team members if needed, for example if a member of the team leaves, is unwell or taking leave.

#### Lesson 6: When working rapidly, there is a need to be responsive to changing needs and circumstances, therefore studies need to be planned to allow for flexibility

This evaluation was conducted in a particularly uncertain time, given the COVID-19 pandemic and the evolving nature of the services that we were evaluating. However, our reflections demonstrate the need for rapid evaluations to develop studies with flexibility to respond to different needs and circumstances relating to team resources, data collection and analysis that may arise. All research evaluations have scope for plans to change or new circumstances to arise, therefore it is imperative to ensure that there is a “plan b” should anything change. Additionally, if the time to scope a study is very short (as with Phase 1 of the evaluation), some of the issues that may have been spotted during scoping may only come to light once the study is underway. Therefore, flexibility is essential as not everything can be agreed or decided upon upfront. Teams therefore need to be comfortable working with emerging and changing circumstances. This recommendation supports previous research which highlights the importance of flexibility in rapid evaluations (Vindrola-Padros et al., 2021a). This is challenging to achieve in practise given that protocols must be specified in advance of conducting the study in order for approvals to be received. Strategies for data collection include planning flexibility into the protocol and procedures (e.g., offering different modes of interviews), and ensuring there is a plan in place for submission of amendments as required. For data analysis, regular discussions are needed to ensure that the planned analyses are still relevant, feasible and appropriate.

### 3.3. Rapid methods

#### Lesson 7: Consider the risks associated with new data collections of quantitative data and their usability

Within mixed-methods rapid research evaluations, it may be necessary to rely on new data collections to evaluate the effectiveness and cost of services. However, as we have described, this can lead to challenges around data incompleteness, poor quality and lack of timeliness. In this evaluation, this was difficult to plan and anticipate in advance, due to the rapidly evolving nature and urgency of COVID-19. However, it is recommended that researchers review the landscape of data as early as possible and assess any risks that may arise and have a back-up plan if the data are ultimately judged to be unusable. Sometimes, as in our study, it may be possible to use surveys to validate new data. In these instances, scoping phases or early phases of the study may be helpful to understand the data landscape.

This, together with Lesson 6 highlight the importance of managing stakeholder expectations and researchers avoiding promising things upfront that they cannot be sure they can deliver on. For example, it may not be clear until some way into a study that a proposed method is not feasible (e.g., our cost effectiveness analysis). Therefore, being honest with stakeholders about Plan A but also alternative plans (Plan B, C and D…), is critical. Within this evaluation, the relationships we built with key stakeholders enabled these open and honest conversations.

#### Lesson 8: Consider whether it is possible to use aggregated quantitative data, and what that would mean when presenting results

Within rapid studies, much, if not all, the quantitative data may only be available at an aggregated level (for example, by site, or by area) rather than at an individual person-level. Project teams therefore need to decide what kinds of quantitative analysis would add value, and present outputs that acknowledge the corresponding degree of precision that is possible. Ranges of uncertainty can be quantified with sensitivity analysis. Such analysis can be important in early feedback to the service and in raising hypotheses that can be taken forward as more detailed data becomes available, or with future evaluations.

#### Lesson 9: Consider using structured processes and layered analysis approaches to rapidly synthesise qualitative findings

Within rapid studies, there are often tensions between completing analyses quickly, and producing publishable analyses. In this study, using structured processes (rapid assessment procedure sheets) helped to ensure that all researchers were following the same approach to summarise findings from interviews, which made high-level data analysis quicker. Additionally, team meetings and regular conversation helped to ensure that all team members completed data analysis tools in largely the same style and method to speed up the process of combining findings from different sites or stakeholders. These high-level data analysis methods, combined with thorough in-depth analyses of particular topics helped to balance speed and academic rigour within this study. This layered approach to analysis also relied heavily on the involvement of many team members in the analysis process and therefore this may require suitable resourcing from a staff perspective.

### 3.4. Team characteristics and management for rapid evaluations

#### Lesson 10: The quicker and more multidisciplinary the study, the larger the team that may be needed (and the more robust the leadership, oversight and management of the team that will be required)

The composition, capabilities and capacity of your evaluation team is a key factor influencing the success of your rapid evaluation. We have shown the importance of ensuring that your rapid evaluation has the following skills and expertise: leadership and management, project management, and a team of researchers with a range of methodological skills and characteristics required to successfully conduct rapid evaluations. For example, a mixed-methods evaluation requires researchers with expertise spanning quantitative, economic, and qualitative backgrounds. Additionally, all of those working on the evaluation will need time available to work on the project. This has been highlighted in previous research which has outlined that one of the challenges to achieving rigour and scope rapidly is the difficulty associated with covering a wide range of questions including access, effectiveness, cost, acceptability, equity and implementation (Norman et al., 2022). We have demonstrated the possibility of covering a large range of topics and questions within rapid evaluations, but that this requires a large team with capacity and skills to do so. Within rapid evaluations, a team-based approach enriches data analysis. Additionally, having a large team of researchers enabled thorough and rapid triangulation of different sources of data (e.g., national quantitative data, health economic data and qualitative data) to rapidly provide a rich evaluation of services.

#### Lesson 11: Ensure that all team members know their roles and responsibilities and have ways of clearly communicating with other members of the team, to ensure that the project continues to progress rapidly

All individuals involved in rapid evaluations should have clear roles and know their responsibilities within these roles. These roles should be agreed on as early as possible within the project, and reviewed as necessary (e.g., in cases of changes to capacity). To support team working there is a need for clear communication channels. Within this evaluation we relied on email, weekly team meetings, and frequent communication *via* MS Teams to ensure that all team members were updated and conduct our evaluation. A shared drive ensured that team members had access to all materials. Whilst there are other modes of communication that could be explored for rapid evaluation (e.g., slack, Trello, and Miro), we did not use these within this evaluation and cannot comment on their utility for rapid research. Clear lines of communication are vital, particularly in rapid projects where there is limited amount of time to catch up if the project falls behind.

#### Lesson 12: Don't slow down or wait when it comes to dissemination. Think about how best to present findings as early as possible so that they can be understood and used quickly (e.g., to make decisions)

Within rapid evaluations, findings must also be disseminated rapidly. Researchers should consider how best to present findings so that they can be understood and used quickly (e.g., to inform decisions). Therefore, it is helpful to provide a dissemination plan or strategy. This plan should include formative feedback throughout the study (e.g., through meetings and analysis workshops), so that external stakeholders are aware of the preliminary findings as early as possible to inform clinical practise. Within rapid studies, it is unlikely that a long, written report will be the dissemination method of choice for external stakeholders, and instead a presentation or slide deck may be more appropriate. Longer reports and academic papers may then come later. The dissemination plan or strategy should include the proposed dissemination activities, target audiences, deadlines for each output and sub-teams who will lead on each output. Within this evaluation, this dissemination plan enabled us to juggle interim and final outputs in a rapid timeframe.

## 4. Summary and conclusions

In summary, this manuscript provides a detailed analysis of our experiences conducting large-scale mixed-methods rapid evaluations of healthcare services implemented during the COVID-19 pandemic. Our reflections on the journey of conducting large-scale rapid evaluations from design through to dissemination provide an insight into the factors that supported and challenged the success of our evaluation for each stage of the research process.

We outline 12 key lessons for conducting large-scale, mixed-methods, rapid evaluations of national healthcare services. We propose that rapid study teams need to: (1) find ways of building trust with external stakeholders quickly, (2) consider the needs of the rapid evaluation and resources needed, (3) use scoping to ensure the study is highly focused, (4) carefully consider what cannot be completed within a designated timeframe, (5) use structured processes to ensure consistency and rigour, (6) be flexible and responsive to changing needs and circumstances, (7) consider the risks associated with new data collection approaches of quantitative data (and their usability), (8) consider whether it is possible to use aggregated quantitative data, and what that would mean when presenting results, (9) consider using structured processes & layered analysis approaches to rapidly synthesise qualitative findings, (10) consider the balance between speed and the size and skills of the team, (11) ensure all team members know roles and responsibilities and can communicate quickly and clearly, and (12) consider how best to share findings for rapid understanding and use.

The reflections and lessons shared within this manuscript may be useful in informing the development and conduct of future robust rapid evaluations. For example, researchers new to the field of rapid evaluation, who are planning on conducting rapid evaluations of health and care services may wish to use our lessons to inform the design and execution of their study, considering important aspects such as stakeholder relationships, leadership, project management and administration, resources, and flexibility.

Further research is needed to consider whether these lessons and reflections extend to large-scale rapid evaluations conducted in non-pandemic/urgent situations.

## Data availability statement

The datasets presented in this article are not readily available because this is a reflective chapter. The findings and data sharing agreements reflected on in this chapter are reported elsewhere. Requests to access the datasets should be directed to NF, n.fulop@ucl.ac.uk.

## Ethics statement

For Phase 1, Phase 2 (effectiveness, cost and staff survey and interviews) and the care homes study, our study received ethical approval from the University of Birmingham Humanities and Social Sciences Ethics Committee (Phase 1: ERNE_13-1085AP37, Phase 2: ERN_13-1085AP39, care homes: ERN_13-1085AP40) and were categorised as a service evaluation by the Health Research Authority (HRA) decision tool and relevant university research governance offices. The Phase 2 patient experience study (survey and case study interviews—workstreams 3 and 4) was reviewed and given favourable opinion by the London-Bloomsbury Research Ethics Committee (REC reference: 21/HRA/0155) (Feb 2021). The patient experience study was categorised as an urgent public health study by NIHR. The patients/participants provided their written informed consent to participate in this study.

## Author contributions

HW drafted the manuscript and finalised the manuscript for submission. All authors commented on, revised the manuscript, discussed the content and structure of this manuscript, including reflections, and key lessons. All authors read and approved the final manuscript.
